# Effect of a Statewide Media Campaign on Smoking Cessation Among Florida Adults

**DOI:** 10.5888/pcd17.190271

**Published:** 2020-02-20

**Authors:** Jennifer C. Duke, Robyn Woodlea, Kristin Y. Arnold, Anna J. MacMonegle, James M. Nonnemaker, Lauren Porter

**Affiliations:** 1 RTI International, Research Triangle Park, North Carolina; 2 Florida Department of Health, Tallahassee, Florida

## Abstract

**Introduction:**

Since December 2010, Florida’s Bureau of Tobacco Free Florida has aired a statewide tobacco education campaign to encourage smoking cessation. The Tobacco Free Florida campaign consists of evidence-based advertisements primarily characterized by strong emotional content and graphic imagery designed to increase awareness of the health risks of tobacco use. We evaluated the effect of the media campaign on population-level quit attempts by using a statewide representative sample of Florida adults aged 18 or older.

**Methods:**

We examined data from 5,418 Florida adult cigarette smokers and recent quitters aged 18 or older from the Florida Adult Tobacco Survey, an annual, cross-sectional survey conducted from 2011 through 2018. The primary outcome was incidence of quit attempts in the previous 12 months. We used multivariable logistic regression models to estimate the odds of making a quit attempt as a function of advertising levels across state media markets. Rates of quit attempts in Florida were also estimated.

**Results:**

Approximately 66% of smokers in the study made at least 1 quit attempt. Exposure to the campaign was associated with increased odds of a quit attempt in the previous 12 months (odds ratio = 1.25; *P* = .02) among smokers and recent quitters. The Tobacco Free Florida campaign was associated with an estimated 332,604 additional smokers making quit attempts per year during the study period.

**Conclusion:**

The Tobacco Free Florida campaign affected cessation-related behaviors in Florida over an 8-year period. Evidence-based state tobacco education campaigns can accelerate progress toward the goal of reducing adult smoking.

**Summary**

**What is already known about this topic?**

Statewide media campaigns are an evidence-based component of comprehensive tobacco control programs. However, because of budget limitations, few state tobacco control programs can devote resources to both create and consistently air media campaigns.

**What is added by this report?**

From 2011 through 2018, the Bureau of Tobacco Free Florida aired hard-hitting advertisements from successful national and state campaigns as part of their Tobacco Free Florida campaign. The study found that exposure to the Tobacco Free Florida campaign influenced the rate of quit attempts among adult smokers in Florida.

**What are the implications for public health practice?**

Statewide media campaigns can use existing effective advertising materials to affect population-level quit attempts.

## Introduction

Research shows that mass media campaigns can effectively encourage smoking cessation and contribute to reductions in smoking prevalence among adults aged 18 or older (1–4). An international review of evidence from 121 anti-tobacco mass media campaigns showed strong evidence of positive behavioral effects (5). However, increases in population-level quit attempts have been shown to fade shortly after a campaign ends (1). A national tobacco education campaign for adults, the Centers for Disease Control and Prevention’s (CDC’s) Tips From Former Smokers (Tips), has aired intermittently since 2012. However, few states have maintained robust paid media campaigns in recent years because of funding constraints (6).

As a result of a ballot initiative, the Florida Constitution was amended in 2007 to allocate funds to a comprehensive statewide tobacco education program. The Florida Department of Health Bureau of Tobacco Free Florida is one of the largest statewide tobacco control programs in the United States. The program’s goal is to protect people from the health hazards of using tobacco by discouraging its use. From 2011 through 2018, the bureau invested approximately $160 million in paid advertising to educate residents about the health risks of tobacco use and the dangers of secondhand smoke exposure. The Tobacco Free Florida media campaign (TFF) primarily used advertisements with highly emotional or graphic content; such content is more likely to be attended to and perceived as effective than less hard-hitting content (7–9). Empirical studies indicate that highly emotional advertisements or those that include graphic imagery are effective in promoting cessation, whereas other types are not as effective (10–12).

The extent to which Florida’s statewide campaign affects behavior change at the population level is a crucial question for both state and federal agencies given their limited resources and the high cost of airing televised mass media campaigns. Our study examined the association between exposure to the state’s anti-tobacco television advertisements and quit attempts among smokers and recent quitters over an 8-year period.

## Methods

### Data source

The Florida Adult Tobacco Survey (13) is an annual cross-sectional survey that assesses tobacco use and behavior among adults aged 18 or older in Florida. The survey uses a random digit dialing method for selecting a new group of participants each year from a landline telephone sample. In 2013, the sampling criteria expanded to include cellular telephone users. The TFF campaign launched in late 2010, and we examined data collected from March 2011 through September 2018 for our study. Of the 48,538 participants from these years, 7,330 current smokers and recent quitters provided information on quit attempts made in the past year and a valid zip code or county of residence. The analysis sample of 5,418 people consisted of participants with complete demographic and smoking-related data used in the analytic models.

Response rates were available from 2013 through 2018 and were as follows: 10.7% for 2013, 9.1% for 2014, 8.8% for 2015, 7.8% for 2016, 8.7% for 2017, and 6.5% for 2018 (14). To produce reliable estimates of the population parameters, we used analysis weights that accounted for unequal probabilities of selection and adjusted for nonresponse. Five sampling distributions were also used to calculate weights that resulted in annual samples representative of the Florida adult population: age, sex, race/ethnicity, educational attainment, and telephone category (cellular telephone, landline, or both).

TFF ran paid advertising on television, radio, the internet, and other outlets from December 2010 through September 2018 to educate Floridians. TFF advertisements contained strong emotional content and graphic imagery designed to increase awareness of the health risks of tobacco use and dangers of secondhand smoke, with the expressed goal of increasing smokers’ interest in quitting. A small number of motivational advertisements complemented these messages by aiming to increase smokers’ self-efficacy to quit and their knowledge of effective cessation practices and services (Table 1).

**Table 1:** Tobacco Free Florida Media Campaign Implementation, Television Advertisements, 2010–2018

Campaign Year	Television Advertisements	Campaign Year Summary
**2010–2011**	Reverse the Damage: Reverse — Heart Attack	The campaign predominantly featured cessation messaging from several campaigns with graphic depictions of the health consequences of smoking and emotional appeals about the impact of smoking on family members. The campaign also included advertisements with secondhand smoke messaging.
	Reverse the Damage: Reverse — Lung Cancer	
	Rick Stoddard: 46 Years	
	Rick Stoddard: Emergency Department	
	(Unspecified Campaign): Apartment	
	Just Eliminate Lies: Baby Seat	
	(Unspecified Campaign): Separation	
	Every Cigarette Does Damage: Artery	
	Every Cigarette Does Damage: Lung	
**2011–2012**	Every Cigarette Does Damage: Artery	The campaign featured a mixture of cessation messaging from several campaigns with graphic depictions of and testimonials about the health consequences of smoking and motivational messaging to offer support and encouragement to quit. The campaign also included advertisements with secondhand smoke messaging.
	Every Cigarette Does Damage: Lung	
	Quitting Takes Practice: I Know, I Know	
	Quitting Takes Practice: Smoker’s Helpline	
	Reverse the Damage: Reverse — Heart Attack	
	Reverse the Damage: Reverse — Lung Cancer	
	Tobacco. Reality. Unfiltered: This is Justin	
	Tobacco. Reality. Unfiltered: This is Destini	
	(Unspecified Campaign): Don’t Stop Fighting	
	(Unspecified Campaign): Apartment	
	Just Eliminate Lies: Baby Seat	
	(Unspecified Campaign): Separation	
**2012–2013**	Tips From Former Smokers: Cessation Tips	The campaign predominantly featured a mixture of cessation messaging from the Centers for Disease Control and Prevention’s Tips From Former Smokers campaign (https://www.cdc.gov/tobacco/campaign/tips/index.html). At the end of the campaign year, the campaign also included advertisements with secondhand smoke messaging.
	Tips From Former Smokers: Anthem	
	Tips From Former Smokers: Suzy's Tip	
	Tips From Former Smokers: Jessica's Asthma Tip	
	Tips From Former Smokers: Roosevelt's Tip	
	Tips From Former Smokers: Buerger's Tips	
	Tips From Former Smokers: Terrie's Tip	
	Reverse the Damage: Reverse — Heart Attack	
	Reverse the Damage: Reverse — Lung Cancer	
	Just Eliminate Lies: Baby Seat	
	(Unspecified Campaign): Separation	
**2013–2014**	Tips From Former Smokers: Terrie's Voice Tip	The campaign predominantly featured a mixture of cessation messaging from the Centers for Disease Control and Prevention’s Tips From Former Smokers campaign.
	Tips From Former Smokers: Buerger's Tips	
	Tips From Former Smokers: Nathan’s Tip	
	Tips From Former Smokers: Cessation Tips	
	Tips From Former Smokers: Bill’s Tip	
	Tips From Former Smokers: Tiffany’s Tip	
	Tips From Former Smokers: Anthem	
	Tips From Former Smokers: Roosevelt's Tip	
	Tips From Former Smokers: Terrie Teenager	
	Become an Ex: Start Your Day	
**2014–2015**	Tips From Former Smokers: Terrie Teenager	The campaign exclusively featured a mixture of cessation messaging from the Centers for Disease Control and Prevention’s Tips From Former Smokers campaign.
	Tips From Former Smokers: Buerger's Tips	
	Tips From Former Smokers: Nathan’s Tip- Memorial	
	Tips From Former Smokers: Terrie Surgeon General	
	Tips From Former Smokers: Bill’s Tip	
	Tips From Former Smokers: Tiffany’s Tip	
	Tips From Former Smokers: Terrie Don’t Smoke	
	Tips From Former Smokers: Brett’s Tip	
	Tips From Former Smokers: Shawn’s Tip	
	Tips From Former Smokers: Amanda’s Tip	
**2015–2016**	Tips From Former Smokers: Terrie Surgeon General	The campaign exclusively featured a mixture of cessation messaging from the Centers for Disease Control and Prevention’s Tips From Former Smokers campaign.
	Tips From Former Smokers: Bill’s Tip	
	Tips From Former Smokers: Roosevelt's Tip	
	Tips From Former Smokers: Cessation Tips	
	Tips From Former Smokers: Amanda’s Tip	
	Tips From Former Smokers: Terrie Teenager	
	Tips From Former Smokers: Buerger's Tips	
	Tips From Former Smokers: Rose’s Tip	
	Tips From Former Smokers: Tiffany’s Decision	
	Tips From Former Smokers: Marlene’s Needle Tip	
	Tips From Former Smokers: Mark and Julia’s Tip	
	Tips From Former Smokers: Jessica's Asthma Tip	
	Tips From Former Smokers: Nathan’s Tip — Memorial	
**2016–2017**	Lost Moments: Hopscotch	The first half of the campaign year predominantly featured motivational cessation messaging from ClearWay Minnesota’s No Judgements campaign (http://clearwaymn.org/tobaccos-harm/campaign-ads/) to promote Tobacco Free Florida’s “Quit Your Way” program (https://tobaccofreeflorida.com/how-to-quit-tobaco/smoking-cessation-programs/) along with some graphic and emotional cessation messaging and secondhand smoke messaging. At the end of the campaign year, the campaign began featuring hard-hitting testimonial cessation messaging from the New York City Department of Health and Mental Hygiene’s Quitting is Hard, Cancer is Harder campaign (https://www1.nyc.gov/site/doh/about/press/pr2016/pr021-16.page) along with motivational cessation messaging from ClearWay Minnesota’s No Judgements campaign.
	Lost Moments: Military Homecoming	
	Lost Moments: We’re Having a Baby	
	Secondhand Smoke Kids: Secondhand Smoke, Kids Under 5	
	Secondhand Smoke Kids: Secondhand Smoke, Kids Under 10	
	No Judgments: Angie	
	No Judgments: Wendall	
	Suffering Every Minute: Lung Cancer	
	Suffering Every Minute: Mom Cancer	
	Suffering Every Minute: Emphysema	
	Quitting is Hard, Cancer is Harder: How Can I Have Lung Cancer?	
	Quitting is Hard, Cancer is Harder: Out of My Hands	
	Quitting is Hard, Cancer is Harder: Field of Radiation	
	Quitting is Hard, Cancer is Harder: Is it Going to Come Back?	
**2017–2018**	Quitting is Hard, Cancer is Harder: How Can I Have Lung Cancer?	The first quarter of the campaign year continued to feature hard-hitting testimonial cessation messaging from the New York City Department of Health and Mental Hygiene’s Quitting is Hard, Cancer is Harder campaign and messaging from ClearWay Minnesota’s No Judgments campaign. In the second quarter of the campaign year, the campaign switched from the Quitting is Hard, Cancer is Harder campaign to hard-hitting testimonial messaging from the Center for Disease Control and Prevention’s Tips From Former Smokers campaign. In the spring of 2018, Tobacco Free Florida launched original motivational advertising from The Reasons campaign (https://tobaccofreeflorida.com/reasons-quit-smoking-stories/).
	Quitting is Hard, Cancer is Harder: Out of My Hands	
	Quitting is Hard, Cancer is Harder: Field of Radiation	
	Quitting is Hard, Cancer is Harder: Is it Going to Come Back?	
	No Judgments: Angie	
	No Judgments: Wendall	
	Tips From Former Smokers: Terrie Teenager	
	Tips From Former Smokers: Amanda’s Tip	
	Tips From Former Smokers: Brian’s Heart Attack Tip	
	Tips From Former Smokers: Roosevelt's Tip	
	Tips From Former Smokers: Rebecca’s Tip	
	The Reasons: Christy	
	The Reasons: Robert	

### Measures

The independent variable of interest in the statistical model was exposure to TFF television advertisements. We measured exposure by using target rating points (TRPs), which are the standard unit of measurement for media delivery (4). TRPs are the product of 2 measures: the percentage of a target population potentially exposed to advertisements (reach) and the average number of times advertisements are aired (frequency) over a time period. For example, if 50% of the target population was exposed to 2 advertisements during a week, then the weekly TRPs value is 100. Because TRPs measure potential exposure rather than actual exposure, they offer the advantage of being exogenous to the respondent and therefore not susceptible to the bias inherent in self-reported measures of exposure. We used weekly TRPs for each of Florida’s 10 designated marketing areas (DMAs) for the target audience of adults aged 18 to 54 to create a past-year cumulative TRPs measure. For each respondent, the TRPs value was the sum of weekly TRPs specific to their DMA of residence for the 52 weeks before their survey date. Past-year TRPs for respondents in the analysis sample ranged from 1,233 to 8,477 TRPs, with an unweighted average of 3,887 TRPs and a weighted average of 4,190 TRPs.

Quit attempts, which mark progress in achieving successful tobacco cessation, are the standard outcome measure of effectiveness for anti-tobacco mass media campaigns. The outcome variable was whether a respondent made a quit attempt during the past year: “During the past 12 months, have you stopped smoking for one day or longer because you were trying to quit smoking?” Interviewers asked this question only if a person reported 1) smoking at least 100 cigarettes in their life and 2) smoking cigarettes “some days” or “every day” at the time of the survey. We included recent quitters, defined as former cigarette smokers who successfully stopped smoking in the past year, in the group of respondents who made a quit attempt in the past year.

Demographic and smoking-related variables included indicators for 4 age categories (age 18–24 was used as the reference); an indicator for male (female was the reference category); indicators for non-Hispanic black, Hispanic, and other non-Hispanic race/ethnicity (non-Hispanic white was the reference category); employment (employed for wages/self-employed versus neither); indicators for 4 educational attainment categories (less than completion of high school was the reference category); the presence of a youth under the age of 18 in the household; and the heaviness of smoking index (HSI). HSI incorporates responses to 2 questions: “On average, on days when you smoked during the past 30 days, about how many cigarettes did you smoke a day?” and “On the days that you smoke, how soon after you wake up do you have your first cigarette?” Each item response had a value between 0 and 3, producing an HSI ranging from 0 to 6. A higher HSI reflects greater nicotine dependence (ie, more cigarettes smoked and smoking soon after waking).

We also used 2 media-related variables to control for time spent watching television and exposure to the CDC’s national Tips advertisements, which intermittently ran concurrent to TFF campaign advertisements. The wording of the item assessing time spent watching television changed twice over the years analyzed. From 2011 through 2015 it read “Please think back to the last 7 days. On average, how many hours per day did you watch television?” In 2016, the item changed to: “Think about how much you watched television yesterday. About how much time did you spend watching television shows or movies on any platform including a television set, a computer, a laptop, a tablet, a smartphone, or an iPod or MP3 player?” In 2017, the item changed to: “About how much time did you spend watching television shows or movies yesterday? Include time spent watching on a television, computer, laptop, tablet, or smartphone.” We created a dichotomous measure based on the median split of responses. We measured potential exposure to Tips advertisements for each respondent by summing Tips TRPs in the DMA of residence for 1 year before the respondent’s survey date.

### Statistical analysis

We used a multivariable logistic regression model to examine the likelihood of making a quit attempt in the past year as a function of past-year TFF campaign TRPs. We divided past-year TRPs by 2,000 to yield estimated odds ratios for the changes in the likelihood of a smoker making a quit attempt in the past year, given a unit increase of 2,000 total TRPs in a media market. Scaling TRPs in this way is a common practice to aid in the interpretation of results when advertisement exposure is the independent variable of interest (10,15). As in other media studies (2), the model included demographic, smoking-related, and media-related control variables known to influence quit attempts. In addition, we included an indicator for the DMA in which respondents resided and a linear yearly time trend. At least 2 potential confounders used in other media studies were unavailable in the survey: diagnosis of chronic health conditions and diagnosis of mental health conditions (16).

As in other assessments of the effect of smoking cessation media campaigns on quit attempts, we included numerous additional variables in the multivariable logistic regression model (2,10,17). Three variables — presence of another smoker in the home, having rules about smoking in the home, and income — were not significantly associated with quit attempts in either bivariate or multivariable models; they were excluded from the final model specification. We tested other functional forms for the TRPs variable (eg, square root, parabolic) in alternate models, but these changes did not increase the model’s goodness of fit. The analysis sample size (N = 5,418) was sufficient for reliably identifying effects at a 5% significance level.

We calculated the percentage of smokers who made quit attempts attributable to the TFF campaign by taking the difference between the actual quit attempt percentage from the analysis sample and the predicted quit attempt percentage in a hypothetical scenario where exposure to the campaign was 0 for each year of the analysis sample. We then applied these differences in quit attempt rates to corresponding annual state populations of smokers in Florida (18). We performed all statistical analyses using Stata MP version 15.1 (StataCorp LLC).

## Results

The sample of 5,418 current smokers and recent quitters in Florida from 2011 through 2018 was evenly split between women and men, with similar unweighted and weighted distributions by race/ethnicity (Table 2). Almost 40% of the sample was aged 55 or older. Unweighted income distributions were similar to weighted estimates. Overall, 66% (95% CI, 64.0%–67.7%) of adult smokers in the sample made a quit attempt in the past 12 months. Potential exposure to the campaign was high, with a mean annual TRPs value of 4,190 for the sample, exceeding CDC guidelines for effective campaign reach for a mass media campaign (6).

**Table 2:** Characteristics of Analysis Sample of Adult Florida Smokers Aged 18 or Older (N = 5,418), Florida Adult Tobacco Survey, 2011–2018^a^

Characteristic	n	Unweighted % (SD)	Weighted % (SD)
**Sex**			
Male	2,815	52.0 (50.0)	58.2 (49.3)
Female	2,603	48.0 (50.0)	41.8 (49.3)
**Age, y**			
18–24	571	10.5 (30.7)	10.9 (31.2)
25–34	822	15.2 (35.9)	19.6 (39.7)
35–54	2,025	37.4 (48.4)	43.0 (49.5)
≥55	2,000	36.9 (48.3)	26.5 (44.1)
**Race/ethnicity**			
Non-Hispanic white	3,691	68.1 (46.6)	67.5 (46.8)
Non-Hispanic black	460	8.5 (27.9)	10.9 (31.2)
Hispanic	847	15.6 (36.3)	17.6 (38.1)
Non-Hispanic other	420	7.8 (26.7)	4.0 (19.5)
**Education**			
Some high school	577	10.6 (30.9)	22.3 (41.6)
High school graduate	1,690	31.2 (46.3)	34.6 (47.6)
Some college	1,891	34.9 (47.7)	30.7 (46.1)
College degree or more	1,260	23.3 (42.3)	12.3 (32.9)
**Employment**			
Employed	2,782	51.3 (50.0)	51.8 (50.0)
Not employed	2,636	48.7 (50.0)	48.2 (50.0)
**Annual household income^b^, $**			
<10,000	477	9.6 (29.5)	11.8 (32.3)
10,000–19,999	855	17.3 (37.8)	19.7 (39.8)
20,000–49,999	2,018	40.7 (49.1)	41.7 (49.3)
≥50,000	1,606	32.4 (46.8)	26.7 (44.3)
**Children in the household aged <18 y**			
Yes	1,689	31.2 (46.3)	35.2 (47.8)
No	3,729	68.8 (46.3)	64.8 (47.8)

Abbreviation: SD, standard deviation.

a Florida Adult Tobacco Survey, 2011–2018 (13).

b Because of nonresponse, n = 4,956.

Results from the multivariable logistic regression model (Table 3) indicated that for each increase of 2,000 past year TRPs, a smoker in Florida was 25% more likely to make a quit attempt during that same year (OR = 1.25, *P* = .02). Hispanic smokers were more likely to make a quit attempt than non-Hispanic white adults. Smokers with children in the household aged younger than 18 years were more likely to try to quit than smokers with no children in the household aged younger than 18 years. Smokers with higher HSI scores were less likely to try to quit than those with lower scores.

**Table 3:** Quit Attempts in the Past Year Among Adult Florida Smokers Aged 18 or Older (N = 5,418) in Response to Potential Exposure to the Tobacco Free Florida Campaign^a^

Independent variables	OR (95% CI)	*P* Value
**Past year TRPs^b^**	1.25 (1.04–1.51)	.02
**Sex**		
Female	1 [Reference]	
Male	0.89 (0.75–1.05)	.16
**Age, y**		
18–24	1 [Reference]	
25–34	0.80 (0.57–1.12)	.20
35–54	0.94 (0.68–1.29)	.70
≥55	0.79 (0.57–1.10)	.16
**Race/ethnicity**		
Non-Hispanic white	1 [Reference]	
Non-Hispanic black	1.07 (0.78–1.45)	.68
Hispanic	1.37 (1.06–1.76)	.02
Non-Hispanic other	1.03 (0.73–1.45)	.88
**Employed**		
No	1 [Reference]	
Yes	0.96 (0.80–1.15)	.63
**Children in the household aged <18 y**		
No	1 [Reference]	
Yes	1.29 (1.07–1.57)	.009
**Heaviness of smoking index^c^**	0.79 (0.74–0.83)	< .001
**Time trend^d^**	1.04 (0.98–1.09)	.18
**Designated marketing area**		
Miami–Ft. Lauderdale	1 [Reference]	
Tallahassee–Thomasville	0.99 (0.60–1.65)	.98
Orlando–Daytona Beach-Melbourne	0.87 (0.61–1.23)	.43
Tampa–St. Petersburg (Sarasota)	1.07 (0.75–1.52)	.72
West Palm Beach–Ft. Pierce	0.90 (0.61–1.34)	.60
Jacksonville	1.08 (0.74–1.59)	.69
Ft. Myers–Naples	0.63 (0.40–0.98)	.04
Gainesville	0.97 (0.52–1.84)	.93
Panama City	0.56 (0.34–0.92)	.02
Mobile–Pensacola (Ft. Walton Beach)	0.70 (0.43–1.15)	.16

Abbreviations: CI, confidence interval; OR, odds ratio; TRPs, target rating points.

a Florida Adult Tobacco Survey, 2011–2018 (13).

b TRPs are the standard media buying metric for television advertisements. TRPs were divided by 2,000 so that the OR is an estimate of the effect of an increase of 2,000 TRPs.

c The heaviness of smoking index incorporates the number of cigarettes smoked per day and how soon after waking a cigarette is smoked. A higher index implies higher nicotine dependence.

d Yearly time trend is a linear variable.

**Figure 1:**
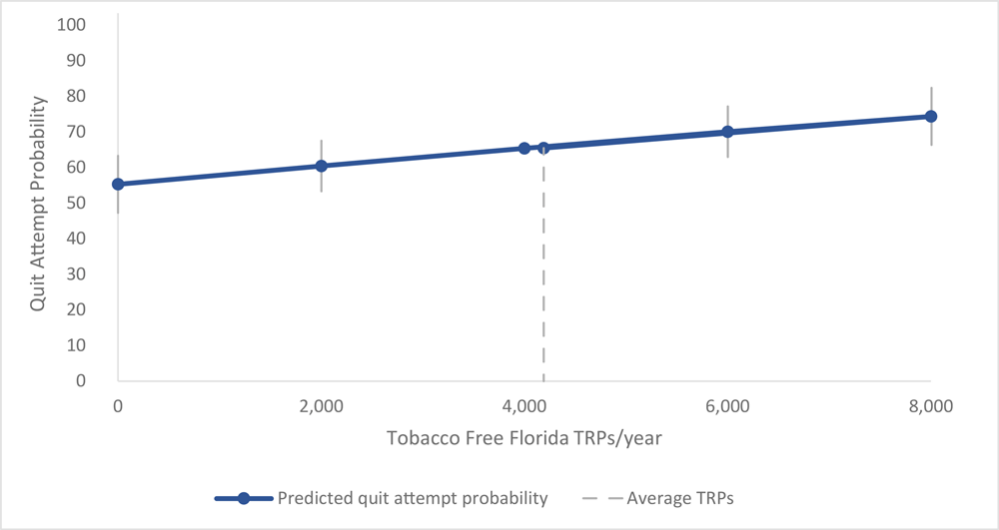
Quit attempt probability as a function of past year Tobacco Free Florida target rating points, Florida 2011–2018. Quit attempt probabilities were predicted by using estimates from a logistic regression model that controlled for age, sex, race/ethnicity, nicotine dependence, children younger than 18 years of age residing in the home, educational attainment, employment status, potential exposure to the Centers for Disease Control and Prevention's Tips From Former Smokers campaign, time spent watching television, media market, and year. Error bars show 95% confidence intervals. The average number of target rating points (TRPs) was 4,190.

By using estimates from the multivariable logistic regression model, we predicted the probability of making a past-year quit attempt at various levels of total annual TFF TRPs. In the hypothetical absence of the campaign, where TRPs were 0, we predicted the overall quit attempt probability for adult Florida smokers to be 55.3% (95% CI, 46.3%–64.4%) (Figure 1). Predicted quit attempt probability increased to 60.5% (95% CI, 54.7%–64.9%) with the first 2,000 TFF TRPs and then continued to increase, but at a diminishing rate, with larger TRP amounts. The predicted quit attempt probability at the average TRP value for the analysis sample (4,190) was 65.9% (95% CI, 63.7%–67.2%).

**Table:** 

Target Rating Points	Predicted quit attempt probability, % (95% Confidence Interval)
0	55.3 (46.3-64.4)
2,000	60.5 (55.8-65.2)
4,000	65.4 (63.7-67.2)
4,190 (Average)	65.5 (63.7-67.2)
6,000	70.1 (66.1-74.0)
8,000	74.4 (67.5-81.2)

**Figure 2:**
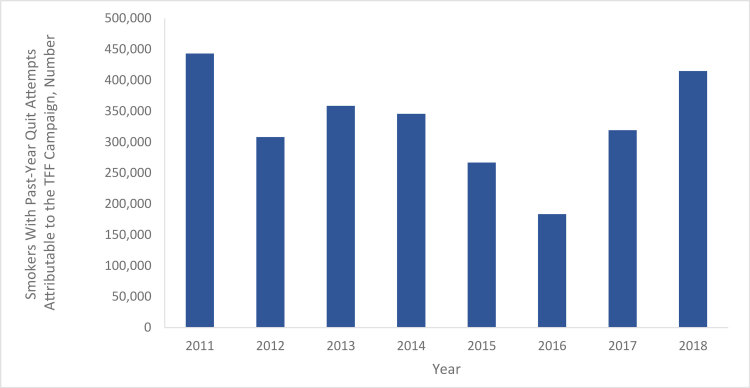
Estimated number of smokers with past year quit attempts attributable to the Tobacco Free Florida (TFF) campaign. These estimates were calculated by taking the annual overall difference between the actual quit attempt percentage for the sample and the predicted quit attempt percentage in a hypothetical scenario where no exposure to the campaign occurred (ie, target rating points = 0), and then applying that difference to the state population of adult smokers in Florida.

To illustrate the influence of the campaign on quit attempts, we estimated the annual number of Florida smokers who made quit attempts attributable to the TFF campaign during the study period. The overall difference between the actual quit attempt percentage for the sample and the predicted quit attempt percentage in a hypothetical scenario where no exposure to the campaign occurred (ie, TRPs = 0) was 10.5 percentage points (95% CI, 1.5–19.6) for the study period, and differences varied from 6.6 to 14.7 percentage points annually across survey years. Applied to the state population of adult smokers in Florida, we estimated that from 2011 through 2018, the TFF campaign was associated with a population weighted average of 332,604 additional smokers making quit attempts per year (or 17% of all smokers who made quit attempts), with annual estimates ranging from 183,369 additional smokers making quit attempts in 2016 to 443,044 additional smokers making quit attempts in 2011 (Figure 2).

**Table:** 

Year	Number of Smokers With Past Year Quit Attempts Attributable to the TFF Campaign
2011	443,044
2012	307,971
2013	358,624
2014	345,607
2015	266,833
2016	183,369
2017	319,240
2018	414,986

## Discussion

Ours is the first study to assess the multiyear impact of Florida’s tobacco education campaign on statewide quit attempts in the context of other nationwide mass media campaigns airing over the past 8 years. We examined the association between temporal and geographic variation in campaign exposure and quit attempts over an 8-year period. Our study used an exogenous measure of media dose, market-level TRPs, which is the most rigorous approach to measurement in natural experiments like media campaigns when control groups are unfeasible (19). Our study suggests that statewide mass media campaigns make an important public health impact by increasing population-level quit attempts, which then increases the number of sustained quitters and reduces smoking prevalence. Data on adult smoking prevalence in Florida during the study period support this conclusion. Adult smoking rates in Florida declined substantially, from 19.3% in 2011 to 14.5% in 2018 (20). These findings also add to the knowledge base for the study of state public health campaigns in real-world settings. Statewide campaigns can complement national tobacco education campaigns that do not air continuously throughout the year nationwide or in Florida.

This study has several limitations. First, the data are cross-sectional and thus show an association between TRPs and quit attempts that is not as strong as in longitudinal designs (2). Second, TRPs are measured at the market level rather than the individual level and thus represent potential as opposed to actual campaign exposure. TRPs are preferred to other available exposure measures (eg, self-reported exposure) because they are exogenous to the individual and not subject to selective attention bias. The use of TRPs may limit the overall variability in the exposure variable and lower statistical power to detect campaign effects. Third, although the model examined exposure to other tobacco-related media campaigns, the multicollinearity we observed with the time trend resulted in a model that may not fully account for the independent or synergistic effects with national cessation campaigns. Fourth, media data for other important media channels such as online advertising could not be included in the exposure variable because of measurement limitations. A final limitation was the relatively low response rates for this study, which may increase the risk of error for estimates. In the past decade, response rates have been declining for national and state surveillance systems (21).

Despite their positive benefits to public health, state tobacco control programs were outspent almost 10 to 1 by the tobacco industry in Florida during the study period (22). State media campaigns like the Tobacco Free Florida campaign are critical to counter tobacco industry marketing and continue to reduce smoking prevalence. In combination with other comprehensive tobacco control efforts, media campaigns can reduce the morbidity and mortality associated with smoking-related diseases in Florida. Estimates indicate that for every pack of cigarettes purchased in Florida, the state spends $12.28 in smoking-related health costs and lost productivity (23). Given the financial burden of health care costs on states, this study suggests that continued funding of state mass media campaigns contributes to reductions in future tobacco use and the resulting public health burden to Florida.
